# How Many Doctors Do We Need in the Public Sector?: A Guide to Human Resource Planning and Specialist Training

**DOI:** 10.21315/mjms2023.30.2.1

**Published:** 2023-04-18

**Authors:** Hirman Ismail

**Affiliations:** Medical Development Division, Ministry of Health Malaysia, Putrajaya, Malaysia

**Keywords:** healthcare system, specialist training, Ministry of Health, human resource for health

## Abstract

Estimating number of doctors including medical specialists needed in the public sector is fundamental to guide human resource planning and implementation of specialist training in Malaysia. Crude population-based and individual basic specialities population-based ratios were used to estimate number of doctors including specialists needed in the public sector by 2025 and 2030. These estimates were then compared with existing number of specialists, current production rates and other parameters to determine level of deficit of the various medical specialities in the future. Medical specialist production versus deficit index was introduced as a tool to present the expected outcome of the existing specialist training. The index can be used as a guide to strategise policies and implementation plans related to training and human resource.

## Introduction

As described by the World Health Organization in 2007, health workforce is one of important building blocks of a health system (1). Development of health workforce shall coincide with development of other building blocks such as service delivery, information, medicine, vaccine and technologies, financing and leadership/governance. It is important that physical development within the Ministry of Health Malaysia such as development of new hospitals and health clinics shall be done in parallel with initiatives to develop human resources for health (HRH). A well-defined roadmap and strategies are needed to ensure sustainability of our healthcare system predominantly provided and maintained by the public sector.

### How Many Doctors Do We Need in the Public Sector?

Secondary data analysis of an open-source World Bank data revealed that in the year 2019, average doctors to population ratio of high-income countries was at 3.3 doctors for every 1,000 population. Upper-middle income countries had 2.2 doctors to 1,000 population, while lower-middle- and lower-income-countries were at 0.7 and 0.5, respectively (2). In 2019, Malaysia had 2.0 doctors for every 1,000 population (3). Such benchmarks are important to estimate number of doctors needed in this country. Quantification of needs of HRH shall preferably be done through predicted workloads rather than population- or facility-based approaches (4), but such approach may take longer time to accomplish and could potentially be resource intensive especially when our primary concern is about defining needs at the national level. A multi-agency workshop on human resource needs in the country was hosted by the then Minister of Health on 2 December 2021 where doctors and other professions ratio to population were tabled and discussed. It was decided that it was appropriate for the Ministry of Health Malaysia to target at least 2.5 doctors for every 1,000 population by 2025 and 3.0 doctors for every 1,000 population by 2030. These translate into 1.0 doctor to 400 population and 1.0 doctor to 330 population by 2025 and 2030, respectively. Consensus was achieved based on benchmarking, capacity to produce, population needs, burden of diseases and health system workload.

**Figure 1:**
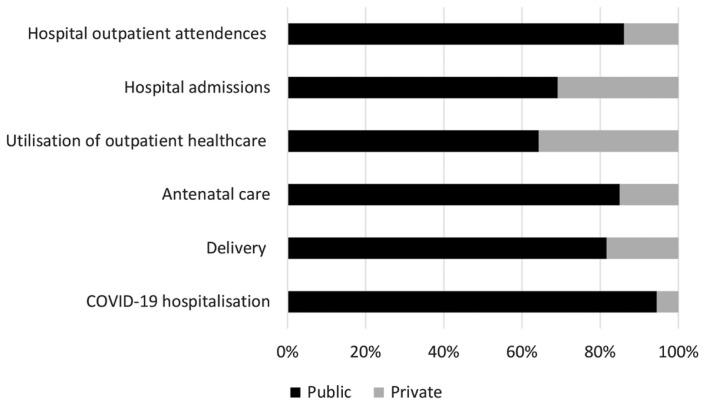
Distribution of burden of the National Healthcare System between the public and private sectors

The Department of Statistics Malaysia (DOSM) has projected that by 2025, the country will be populated by 36,022,700 people and 38,062,200 by the year 2030. Based on ratios as discussed above, Malaysia therefore needs 90,057 actively practicing doctors in 2025 and 114,187 in 2030. The next question would be, how many of these doctors shall serve the public sector? The Malaysian healthcare system is a dichotomous system (or two-tiered) (5) where participation of private sector to the financing and delivery of service is significant. Distribution of HRH in the country shall take into account the needs of both sectors and the level of their overall contribution to the system. Secondary data analysis of multi-source database—Malaysia’s Health Facts, Health Informatic Centre, National Health Morbidity Survey and Medical Programme Information System—was done to examine distribution of major workload of the national healthcare system. When compared to the private sector, the public sector takes up in between 64% and 94% of total major workload which include hospital admissions, outpatient attendances, antenatal visits, deliveries and COVID-19 hospitalisations (6–8), as showed in Figure 1. The apparent discrepancies of distribution of resources including human resource and distribution of workload between the public and private sectors is not new.

Hypothetically at least 70% of HRH distribution shall be at the public sector due to huge workload in government hospitals and clinics, but in many aspects, the distribution is the reversed especially with regards to employment of highly skilled healthcare workers. If we translate the 70/30 distribution of doctors between the two sectors into numbers, the public sector requires 63,040 and 79,931 doctors in 2025 and 2030, respectively, to man health facilities nationwide. It is expected that over the next few years, the workload distribution between these two sectors will remain the same until and unless there’s a major shift in health system reform. It is known that the Government has attempted to spearhead the reform through many initiatives over the years. Latest being, the proposed development of Health White Paper and formation of Health Reform Commission (9, 10); a move to ensure sustainable political will to address the long term and changing needs of our healthcare system. It is hoped that any future reform will help to balance up the maldistribution of resources and burden of the system across all sectors in the country. Until such reform happens, the public sector will continue to remain as the main service provider in the national health system.

### Specialist Training

We examined open- and multi-source database including health authorities’ websites and found that, in developed countries, such as Singapore, Japan, Canada, United States, Australia and United Kingdom, proportion of specialist doctors compared to non-specialist doctors ranges between 41% and 60% (11–16). These advanced countries have considerably high proportion of specialists among general physicians, and these include family physicians in primary care who are also regarded as specialist physicians in those countries. Within the Ministry of Health Malaysia, there are now 8,953 specialists serving the various Ministry’s facilities in 26 basic speciality areas including public health and family medicine. It represents 15.7% of total doctors serving the Ministry. Three-year average of specialists completing gazettement process is currently at 1,073 per year. Gazettement of medical specialists is a formal probation period of at least 6 months that is mandatory to all doctors upon completion of specialist training, following which the person will then be appointed as specialist by the Director General of Health under the General Orders. Names of all appointed specialists are published periodically through the Federal Government Gazette. Over the last 5 years, doctors gazetted as specialists have increased on average 16% annually compared to immediate previous years. The existing specialist training have steadily increased number of specialists needed in the public sector. Based on the current pace of production, the Ministry may achieve approximately 19% of total doctors are specialist doctors by the year 2025, adjusted by average 10% attrition rate (note: attrition rate may differ from speciality to speciality).

How many specialists do we need in the public sector? Estimates of total number of specialists needed in the public sector was also tabled and discussed in the 2 December 2021 workshop as mentioned previously. It was proposed that the public sector needs at least 30% of total doctors in the public sector shall be specialist doctors, that means we need around 18,912 and 23,979 specialists in the year 2025 and 2030, respectively (crude population-based estimates). As mentioned, based on the current production rate, we may only achieve 19%, instead of 30%. The Medical Development Division of the Ministry of Health Malaysia has conducted a series of engagement with heads of clinical services and senior clinicians prior to 2021 to determine appropriate specialists to population ratio by each basic speciality. Through international benchmarking, review of literature and expert opinion, a set of targets was determined. They have been streamlined and adjusted internally, considering capacity to train, production rates and needs for specialist services. Some of these targets may be consistent with achievements of developed countries but some may be set at a lower bound.

Basic speciality areas as listed in Table 1 are based on the Malaysian Medical Council’s list of recognised basic specialities. Pathology however, even though the speciality has been recognised individually as basic specialities—Anatomical Pathology, Chemical Pathology, Genetic Pathology, Haematology and Medical Microbiology—for the purpose of discussion in this paper, the target has been combined. Another exercise is needed to determine their individual targets.

Total number of specialists needed in the public sector can be estimated using the pre-determined individual basic specialities population-based targets or estimates. Considering at least 70% of specialists shall serve the public sector as what proposed previously, it is estimated that the public sector requires 16,792 specialists in 2025 and 19,714 specialists in 2030. These individual basic specialities population-based estimates yield a difference of around 11%–18%, compared to the crude population-based estimates discussed before.

Medical specialist production versus deficit index is proposed as a monitoring tool to track performance of specialist training against the needs of individual specialities. The index is a composite of several data points that compares current deficits of specialists, based on estimates of needs by time or year, with expected production of specialists within given period of time. The index can be summarised as follow:

Medical specialist production versus deficit index in 2023 [individual speciality] by 2030


$$\begin{array}{l} =\frac{\text{expected production by }2030}{\text{current deflict}}\times 100\%\\ =\frac{\left(3\text{-year average annual production}\times 8\;\text{years}\right)}{\left(\text{estimate of number of specialists needed by }2030\text{-current number of specialist}\right)}\times 100\% \end{array}$$

**Figure 2:**
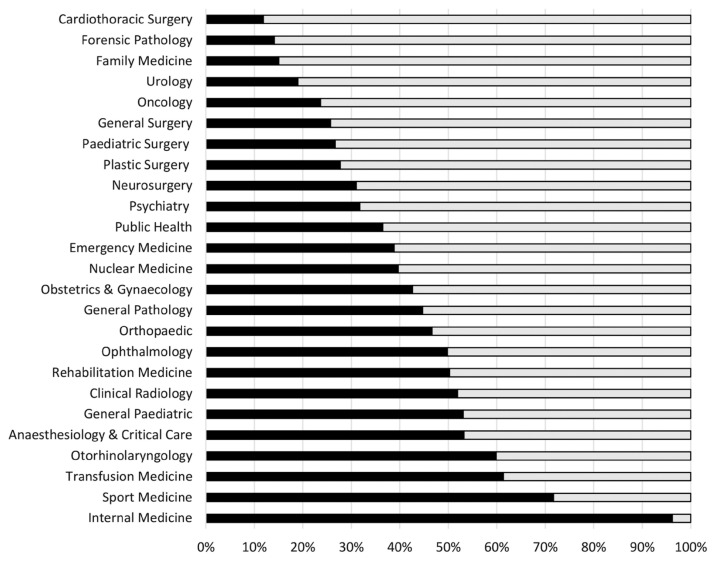
Medical specialist production versus deficit index in 2023, by 2030

The index is not complicated. In the above formula, 2030 was chosen as the based year. Expected production by the year 2030 can be estimated by averaging annual number of specialists gazetted by the Director General of Health in the recent 3 years and multiplied by 8 years (duration from 2023 to 2030). Current deficit can be estimated by substracting current number of specialists in 2023 from estimates number of specialists needed by 2030, based on individual basic specialities population-based estimates as discussed above. The estimates have been adjusted by attrition rate, in this case, 10% was used. Speciality areas with lower index would mean that the existing production rate through the existing training pathways would not be able to address the current quantified deficit of specialists. In other words, the current production would not be able to meet the pre-defined needs for that speciality. Thorough review shall be done on these critical areas to dissect the root cause for such a low index and several strategies or policy direction can be considered as options; for example, increasing local training capacity, overseas training, employment of retired government consultants as trainers, employment of foreign specialists as trainers, incentivise existing specialists to become trainers, partnership with private consultants and institutions and collaboration with international professional bodies. Medical specialist production versus deficit index in 2023 for basic speciality areas listed in Table 1 are shown in Figure 2.

Composite statistics such as the proposed index can be influenced by the pre-defined parameters. Definition of needs for example is a dynamic exercise and can be influenced by many factors including increasing disease burden, population demands, government’s capital investments on facilities, equipment and consumables, development of supporting human capital such as nurses and allied health professionals and many others. Political will and health system reform agenda can also be a strong contributing factor to the pre-defined parameters. What has been defined today may not necessary be the same in the next 2–3 years. The index itself is dynamic. What has been tabled here only addresses the need to develop basic specialities and has not discussed about the needs to develop subspecialty areas that stem from these basic areas. Certain specialities may require more productions compared to the other because of the need to further train a portion of these specialists to more advance subspecialty areas. Training structure for subspeciality areas are different from speciality areas. The needs largely depend on the Ministry’s speciality and subspeciality framework and the ability of the system to provide physical infrastructure and other supporting staff. This shall be discussed separately.

## Conclusion

**Figure 3:**
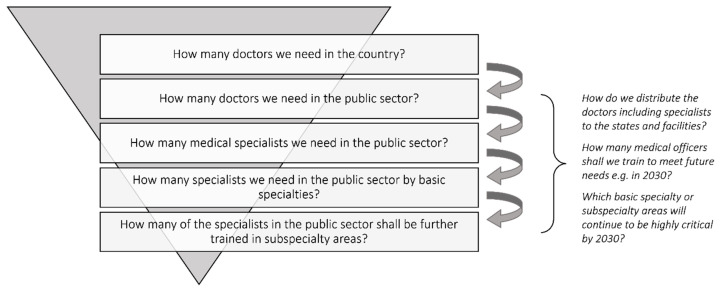
Logic of estimating number of doctors and specialists needed in the public sector by 2025 and 2030

Ensuring adequate supply of HRH especially medical doctors and specialists is pivotal to long term sustainability of the national healthcare system. The health system will continue to grow thus the need to ensure adequate human resource. There are, at present, 44,019 hospital beds within the Ministry of Health system and with the development of several new hospitals and expansion of existing hospitals, number of hospital beds are expected to increase by at least 5,600 beds by the year 2030. At the same time, burden of disease and workload will also grow and potential crisis or outbreak in the future may be more complex than what we experienced with COVID-19. Volume, magnitude and complexity of future challenges warrant the system to be more prepared by ensuring HRH supply in all categories are adequate and appropriate. Principles and logic of estimates using population-based approach as discussed in this editorial is summarised in Figure 3. Such method shall continuously be enhanced using latest evidence and data points. A more refined methodology using predicted workloads or facility-based estimates can be developed to provide better insight on the national needs. Necessary measures shall be employed to address the identified gaps in the provisions of specialist care. Apart from ensuring supply, quality and standards of training deserve the same focus and prioritisation.

## Figures and Tables

**Table 1:** Medical development division’s estimates on specialists to population targets, by Basic Specialities, by 2025 and 2030

Basic speciality	2025	2030	Basic speciality	2025	2030
Anaesthesiology and Critical Care	1 to 20,000	1 to 18,000	Ophthalmology	1 to 40,000	1 to 36,000
Cardiothoracic Surgery	1 to 500,000	1 to 450,000	Orthopaedic	1 to 30,000	1 to 27,000
Clinical Radiology	1 to 30,000	1 to 27,000	Otorhinolaryngology	1 to 70,000	1 to 63,000
Emergency Medicine	1 to 30,000	1 to 27,000	Paediatric Surgery	1 to 250,000	1 to 225,000
Family Medicine	1 to 10,000	1 to 9,000	Pathology	1 to 30,000	1 to 27,000
Forensic Pathology	1 to 200,000	1 to 180,000	Plastic Surgery	1 to 250,000	1 to 225,000
General Paediatric	1 to 20,000	1 to 18,000	Psychiatry	1 to 40,000	1 to 36,000
General Surgery	1 to 20,000	1 to 18,000	Public Health	1 to 25,000	1 to 22,500
Internal Medicine	1 to 11,000	1 to 9,900	Rehabilitation Medicine	1 to 180,000	1 to 162,000
Neurosurgery	1 to 150,000	1 to 135,000	Sport Medicine	1 to 350,000	1 to 315,000
Nuclear Medicine	1 to 300,000	1 to 270,000	Transfusion Medicine	1 to 250,000	1 to 225,000
Obstetrics and Gynaecology	1 to 25,000	1 to 22,500	Urology	1 to 300,000	1 to 270,000
Oncology	1 to 150,000	1 to 135,000			
